# Identification of Reference Genes and Their Application in Analysis of Ginsenoside Biosynthesis Genes in American Ginseng Under Abiotic Stresses and Hormone Treatments

**DOI:** 10.3390/plants15182778

**Published:** 2026-09-10

**Authors:** Fangzhou Zhao, Jiaxin Zhang, Yujuan Zhang, Qingshuai Liu, Debao Huang, Yanwei Zhu, Jinlong Han, Xianchang Wang, Feng Zhang, Shuang Wu, Beijing Tian, Chenggang Shan

**Affiliations:** 1 Institute of Industrial Crops, Shandong Academy of Agricultural Sciences, Jinan 250100, China; 2 Policy and Reform Section, Bureau of Agriculture and Rural Affairs of Weihai Municipality, Weihai 264200, China; 3 Department of Horticultural Science, North Carolina State University, Raleigh, NC 27607, USA

**Keywords:** *Panax quinquefolius*, qRT-PCR, reference gene, expression stability, ginsenoside biosynthesis, abiotic stress

## Abstract

Quantitative real-time PCR (qRT-PCR) is a dominant technique for gene expression analysis in medicinal plants, and accurate qRT-PCR data normalization strictly relies on stably expressed reference genes (RGs). However, systematically validated RGs for *Panax quinquefolius* L. across diverse tissues, abiotic stresses, and hormone treatments are still lacking. In this study, 15 candidate RGs with FPKM values ranging from 7.21 to 210.56 were selected from RNA-Seq datasets based on expression abundance, coefficient of variation, and homology to commonly used reference genes. The expression stability of candidate RGs was evaluated under tissue-specific, drought, waterlogging, gibberellin A3 (GA3), and melatonin (MT) treatments using ΔCt, geNorm, NormFinder, BestKeeper, and RefFinder methods. *UBC28* and *EF1-α* ranked as the top two most stable reference genes across all tested conditions, with stability values of 2.21 and 2.38, respectively. Subsequent qRT-PCR validation of eight ginsenoside biosynthesis-related genes using the *UBC28* and *EF1-α* normalization system showed that drought and waterlogging stresses induced significant expression fold changes (1.5–4.8-fold) in most ginsenoside synthetic genes, while exogenous GA3 and melatonin treatments caused no significant transcriptional regulatory effects under the tested experimental conditions. This study provides a set of reliable, condition-adaptive RGs for *P. quinquefolius* and establishes a robust molecular tool for future studies on ginsenoside biosynthesis and stress response mechanisms in American ginseng.

## 1. Introduction

*Panax quinquefolius* L., commonly known as American ginseng, is a perennial herbaceous species endemic to the deciduous woodlands of eastern North America. It has long been valued in traditional medicine because of its diverse pharmacological activities and is widely used as a natural herbal remedy worldwide [1]. Market demand for *P. quinquefolius* remains strong due to its complex chemical composition and diverse biological activities [2,3]. Most studies on this species have focused on its underground organs, especially saponins, the primary bioactive compounds responsible for its medicinal properties [4]. The biosynthesis and accumulation of these bioactive metabolites are tightly regulated by environmental factors and endogenous hormones via complex transcriptional regulatory networks [5]. Therefore, accurate analysis of gene expression is essential for elucidating gene functions and the molecular regulatory mechanisms governing metabolite biosynthesis, development, and stress responses in *P. quinquefolius* [6,7].

Common approaches for transcript abundance detection include quantitative real-time reverse transcription PCR (qRT-PCR), RNA-Seq, microarray analysis, expressed sequence tags (ESTs), and Northern blotting [8,9]. Currently, the combined strategy of RNA-Seq screening and qRT-PCR validation has become a widely adopted strategy for systematic reference-gene identification in various plant species [10,11,12,13,14,15,16,17,18,19]. The accuracy and reproducibility of qRT-PCR quantification depend primarily on primer specificity, optimized reaction conditions, and appropriate normalization using stably expressed reference genes (RGs) [20,21]. The Minimum Information for Publication of Quantitative Real-Time PCR Experiments (MIQE) guidelines explicitly recommend validating stable RGs for specific experimental conditions, thereby minimizing quantitative bias and ensuring reliable qRT-PCR data [22].

Commonly used housekeeping genes, including *GAPDH*, *ACT*, *UBQ*, *18S rRNA*, *EF1-α*, *CYP*, *TUB*, *SAND*, *eIF-4a*, *TIP41*, and *TUA*, have been widely used as RGs for the normalization in plant gene expression analysis. The Internal Control Gene (ICG) database (v2.0, https://ngdc.cncb.ac.cn/icg/, accessed on 16 March 2025) systematically collects experimentally validated RGs for qRT-PCR normalization [23]. However, increasing evidence indicates that stability of housekeeping genes is condition-dependent [24,25,26,27,28,29]. Accordingly, systematic validation of candidate RGs is essential before their application to ensure accurate normalization of gene expression in specific experimental systems [30,31,32]. Multiple authoritative statistical algorithms, including the ΔCt method [33], geNorm [34], NormFinder [35], BestKeeper [36], and the integrative tool RefFinder [37], have been widely used to evaluate the expression stability of candidate RGs and identify the most suitable RGs for qRT-PCR normalization.

In this study, we integrated transcriptome data mining and systematic qRT-PCR validation to screen stable RGs for *P. quinquefolius*. Fifteen candidate RGs were initially selected from multi-tissue RNA-Seq datasets based on their expression profiles and functional annotation in the ICG database. qRT-PCR assays were optimized for all candidate genes, and their expression stability was systematically evaluated across five experimental conditions, including different tissues, drought, waterlogging, GA_3_, and melatonin treatments, using five widely accepted analytical algorithms. Combined with RefFinder comprehensive ranking and geNorm optimal quantity analysis, we identified both universally stable and condition-specific optimal RGs for gene expression normalization in *P. quinquefolius*. The reliability of the selected RGs was further validated by analyzing the expression patterns of eight key genes involved in ginsenoside biosynthesis. To our knowledge, this is the first comprehensive study to systematically identify and validate RG for qRT-PCR normalization in American ginseng, filling a critical methodological gap for this species. These findings establish a reliable accurate gene expression analysis and provide a robust molecular foundation for future studies on ginsenoside biosynthesis and stress-response regulatory networks in *P. quinquefolius*.

## 2. Results

### 2.1. Screening of Candidate Reference Genes

**Figure 1:**
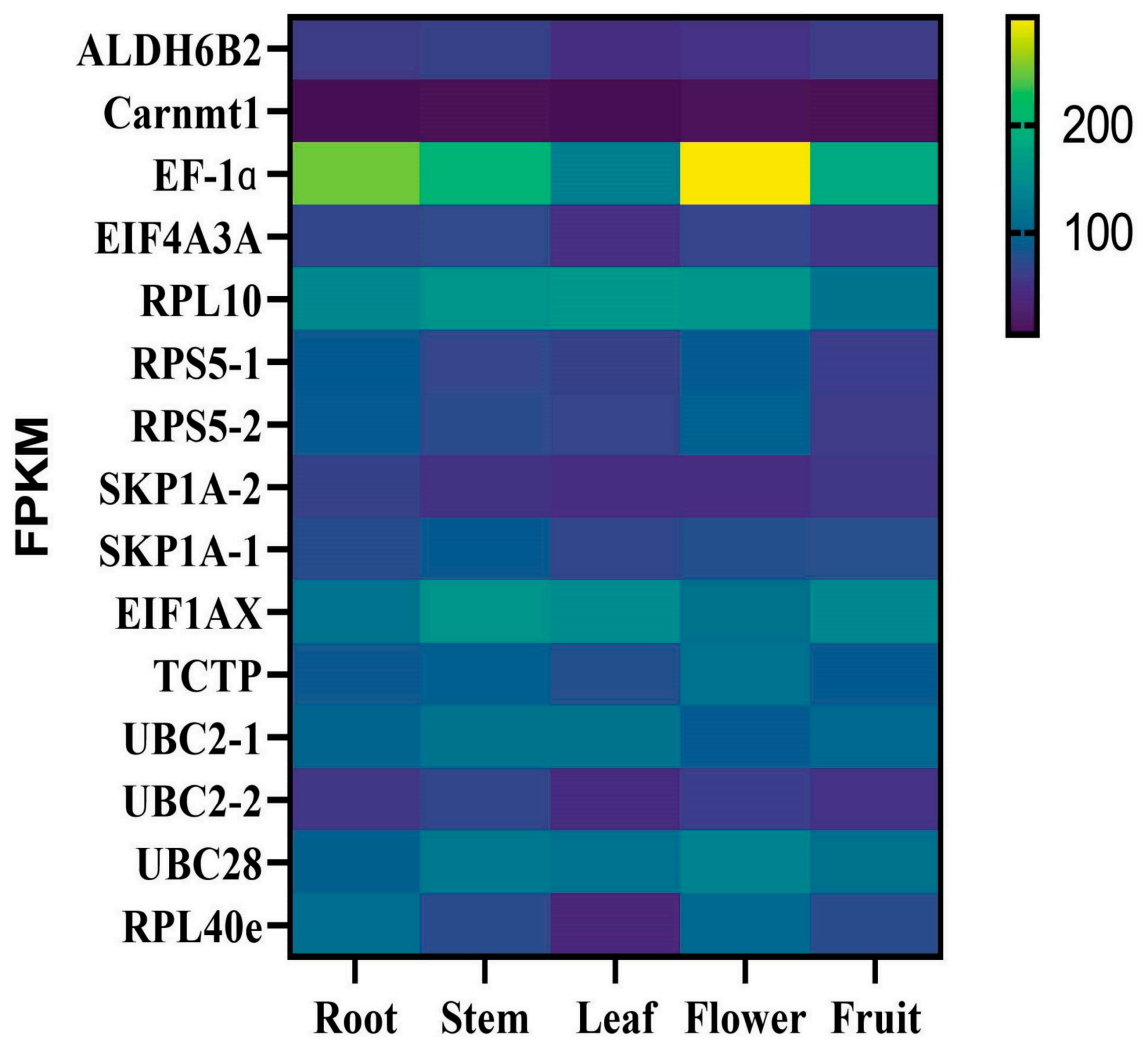
Expression profiles of 15 candidate reference genes in different tissues of *Panax quinquefolius*. The heatmap displays the FPKM (Fragments Per Kilobase of transcript per Million mapped reads) values of each gene across five tissues (Root, Stem, Leaf, Flower, Fruit). The color scale from purple (low FPKM/low expression) to yellow (high FPKM/high expression) represents the range of transcript abundance.

Fifty-five candidate genes were mined from RNA-seq datasets covering five tissues (root, stem, leaf, flower, fruit) using the criteria of mean value (MV) > 50, mean fold-change (MFC) < 1.5, and coefficient of variation (CV) < 0.2. Among them, 12 genes involved in core cellular processes were selected as preliminary candidates based on functional annotation. Additionally, three further candidates (*Carnmt1*, *RPL40e* and *EF1-α*) were screened according to their homologous genes in *Arabidopsis thaliana*. Finally, 15 candidate RGs were obtained for further verification, including several traditional housekeeping genes such as *EF1-α*, ubiquitin-conjugating enzyme genes (*UBC2-1*, *UBC2-2*, *UBC28*), and ribosomal protein-coding genes (*RPL10*, *RPS5-1*, *RPS5-2*, *RPL40e*). The remaining candidates (*ALDH6B2*, *Carnmt1*, *EIF4A3A*, *SKP1A-1*, *SKP1A-2*, *EIF1AX* and *TCTP*) were novel reference-gene candidates mined from multi-tissue transcriptome datasets, as shown in Table S1; Figure 1. Across all analyzed tissues, their FPKM values varied widely from 7.21 to 210.56, and most candidates presented moderate to high transcript abundance suitable for qRT-PCR detection. The tissue expression CV values of these 15 candidates ranged from 0.083 to 0.351; genes such as *SKP1A-1 UBC2-1* and *RPL10* showed lower CV values and exhibited more stable basal expression at the transcriptome level. Preliminary evaluation based on the coefficient of variation (CV) indicated that all candidate RGs exhibited relatively stable expression across tissues, providing a foundation for subsequent stability assessment under abiotic stress and hormone treatments.

### 2.2. Specificity and Optimization of qRT-PCR Primers

**Figure 2:**
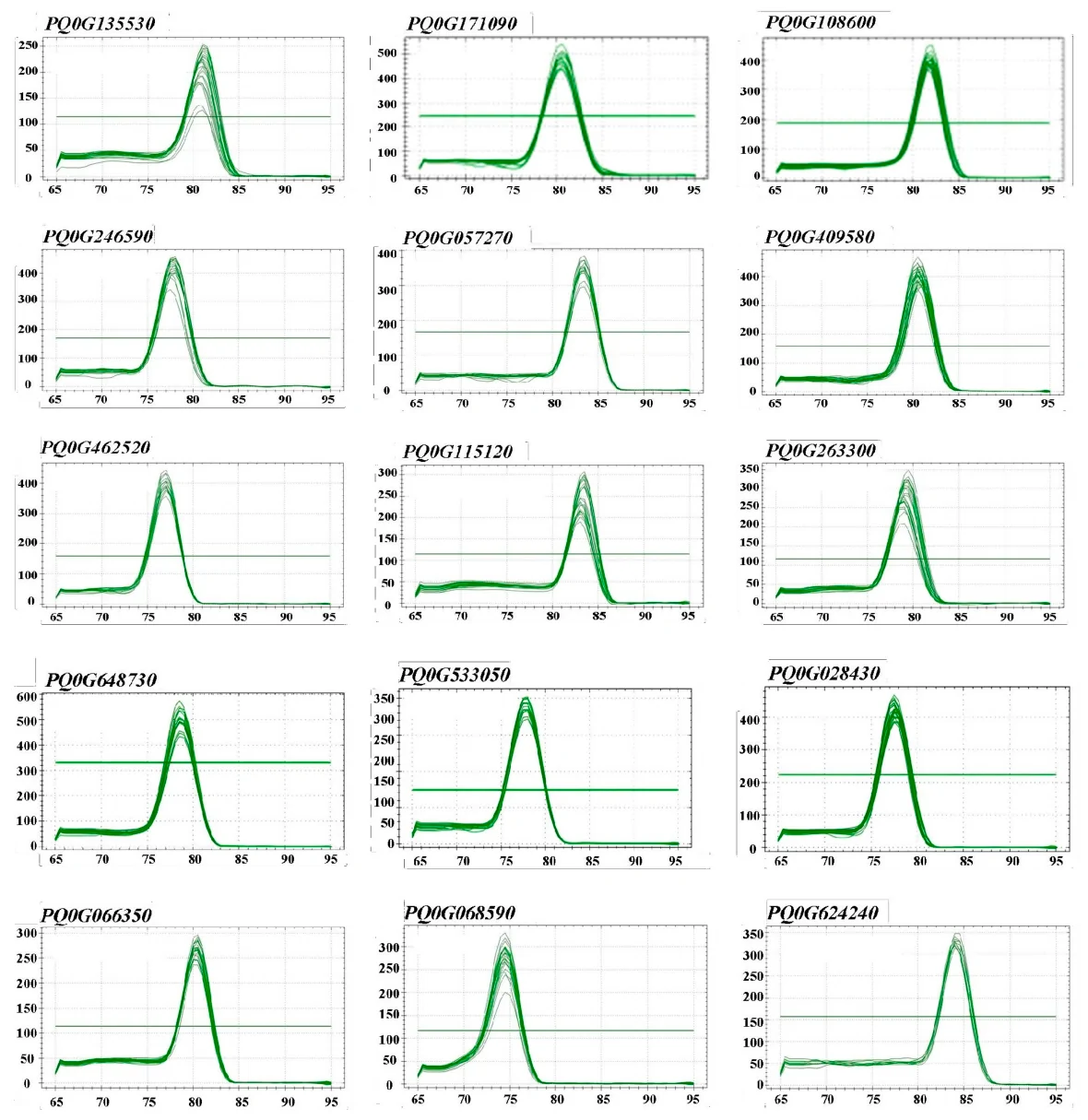
Melting curve of 15 candidate reference genes. The single, sharp peak observed for all genes indicates the amplification of a single specific product.

**Figure 3:**
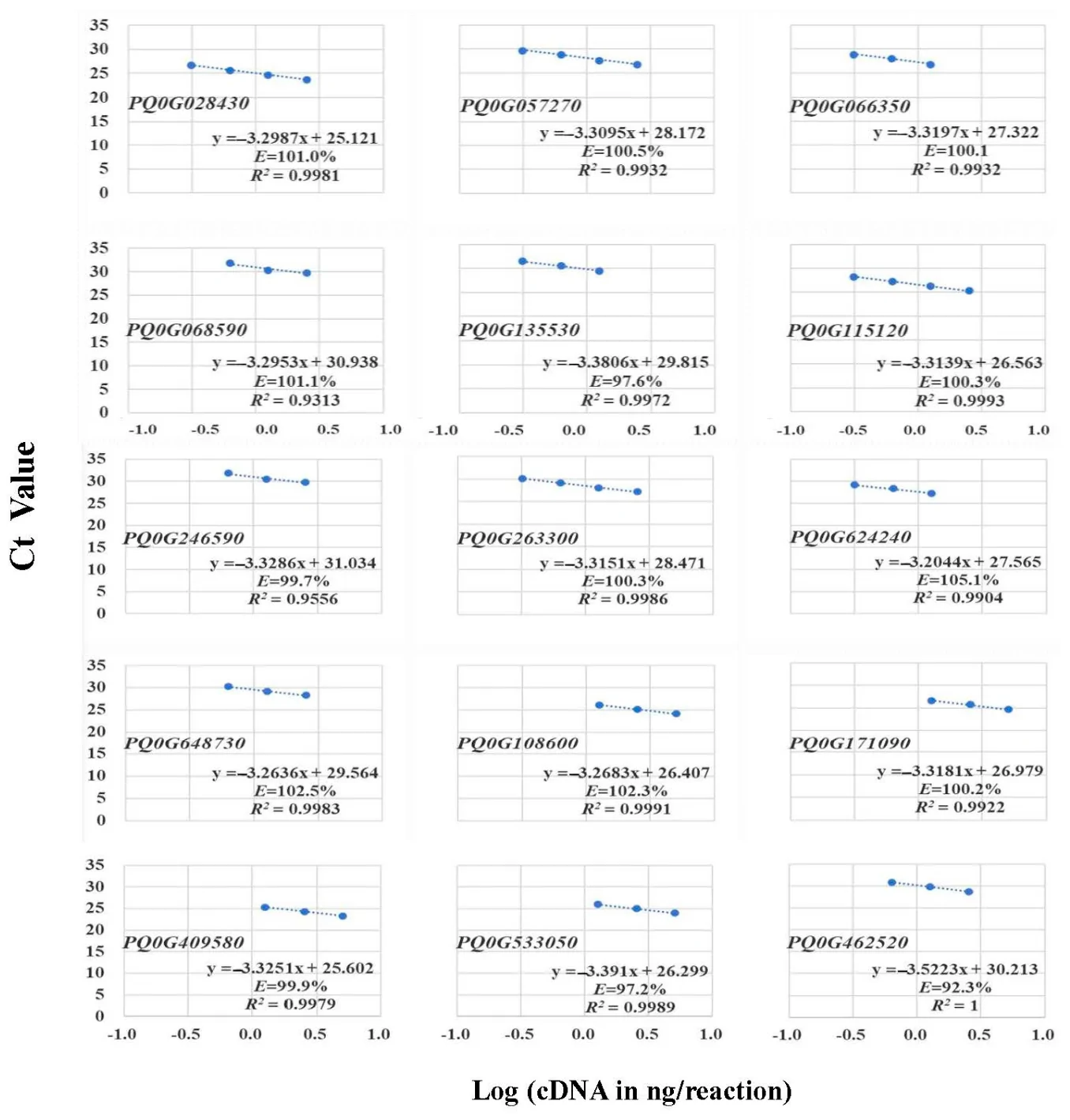
Standard curves for 15 candidate reference genes in qPCR assays. Each panel displays the linear regression (blue line) of Ct values against the log_10_-transformed cDNA template concentration (ng/reaction). The corresponding linear equation, amplification efficiency (*E*, %), and coefficient of determination (*R*^2^) are shown for each gene.

All designed primer pairs met the standard requirements for qRT-PCR analysis, as shown in Table S2. Melting curve analysis confirmed single specific amplification peaks for all optimized primer pairs, with no evidence of non-specific amplification or primer dimer formation, as seen in Figure 2. After gradient optimization of temperature, primer concentration, and template dosage, unqualified primer pairs with abnormal amplification efficiency or low linear correlation were eliminated, as shown in Figure 3. The final optimized primer pairs for all candidate genes exhibited high linear correlation (*R*^2^ ≥ 0.99) and qualified amplification efficiency (100 ± 5%), fully complying with MIQE quantitative standards, as shown in Table 1.

### 2.3. Transcript Abundance Characteristics of Candidate Reference Genes

**Figure 4:**
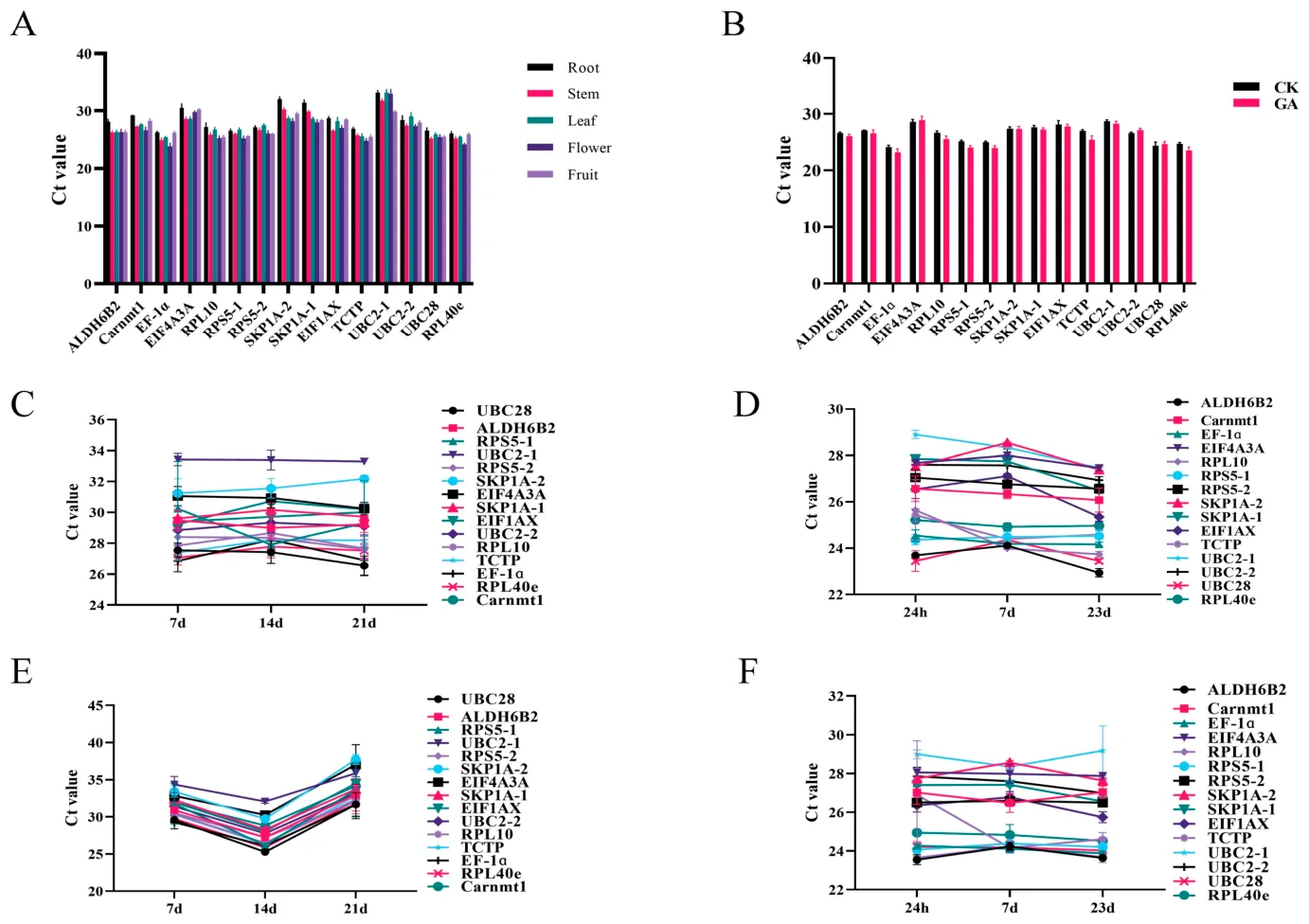
Expression profiles of 15 candidate reference genes across different experimental conditions in *Panax quinquefolius.* The Ct values of each gene were measured under five conditions: (**A**) different tissues (Root, Stem, Leaf, Flower, Fruit); (**B**) GA treatment; (**C**) drought stress at 7, 14, and 21 days; (**D**) melatonin (MT) treatment including the control (CK); (**E**) waterlogging stress at 7, 14, and 21 days; and (**F**) time-course of MT treatment at 24 h, 7 d, and 23 d. Each point represents the mean Ct value ± standard deviation (SD) of three biological replicates. The range and variation in Ct values reflect the expression stability of each gene under the corresponding condition.

The Ct values of candidate RGs ranged from 25 to 32 across all samples, indicating moderate transcript abundance suitable for qRT-PCR normalization. Most candidate RGs exhibited relatively stable expression across different tissues and under drought, GA_3_, and melatonin treatments, as seen in Figure 4. In contrast, long-term waterlogging stress (14 and 21 d) resulted in consistently higher Ct values of all candidate RGs, suggesting an overall reduction in transcript abundance and RNA accumulation under this condition. These results highlight the substantial impact of long-term waterlogging on reference-gene expression and underscore the importance of validating stable, condition-specific RGs before gene expression normalization.

Ct value CV statistics revealed distinct condition-dependent differences in the expression stability of the candidate RGs Table S3. Tissue-specific screening identified *UBC28* and *RPS5-1* as the most stable genes, whereas *UBC2-1* exhibited the highest stability under both drought and waterlogging stresses. Under GA_3_ treatment, *SKP1A-1* and *SKP1A-2* ranked as the most stable RGs, while *RPS5-1* and *RPS5-2* exhibited the most stability under melatonin treatment. These findings demonstrate that the expression stability of reference genes is highly dependent on experimental conditions, highlighting the importance of selecting appropriate condition-specific RGs for accurate qRT-PCR normalization.

### 2.4. Comprehensive Stability Evaluation of Candidate Reference Genes

Independent analyses using different algorithm revealed minor discrepancies in the stability rankings of individual reference genes, which is a common outcome resulting from different statistical principles underlying each method (Table 2 and Table S4, Figure S1). To minimize algorithm-specific bias, the comprehensive ranking generated by RefFinder was used to integrate the results from all four algorithms [38]. This analysis identified *UBC28* and *EF1-α* as the most stably expressed reference genes across all experimental conditions, as seen in Table 2 and Figure S1. In contrast, *RPS5-1* and *UBC2-1* showed the poorest comprehensive stability and were therefore unsuitable as general reference genes for general expression normalization, as shown in Table 2 and Figure S1.

Subgroup analysis further identified high-adaptability condition-specific RGs to meet different experimental requirements: *RPS5-1* and *TCTP* for multi-tissue samples, *UBC28* and *RPS5-2* for drought stress, *UBC28* and *ALDH6B2* for waterlogging stress, *EF1-α* and *RPS5-2* for melatonin treatment, and *UBC2-1* and *ALDH6B2* for GA_3_ treatment (Table 3 and Table S5, Figure S2). Although several reference genes exhibited superior stability under specific experimental conditions, *UBC28* and *EF1-α* maintained the most stable across nearly all conditions, highlighting their universal applicability as universal reference genes for *P. quinquefolius*, as shown in Table 3 and Table S5, and Figure S2.

**Figure 5:**
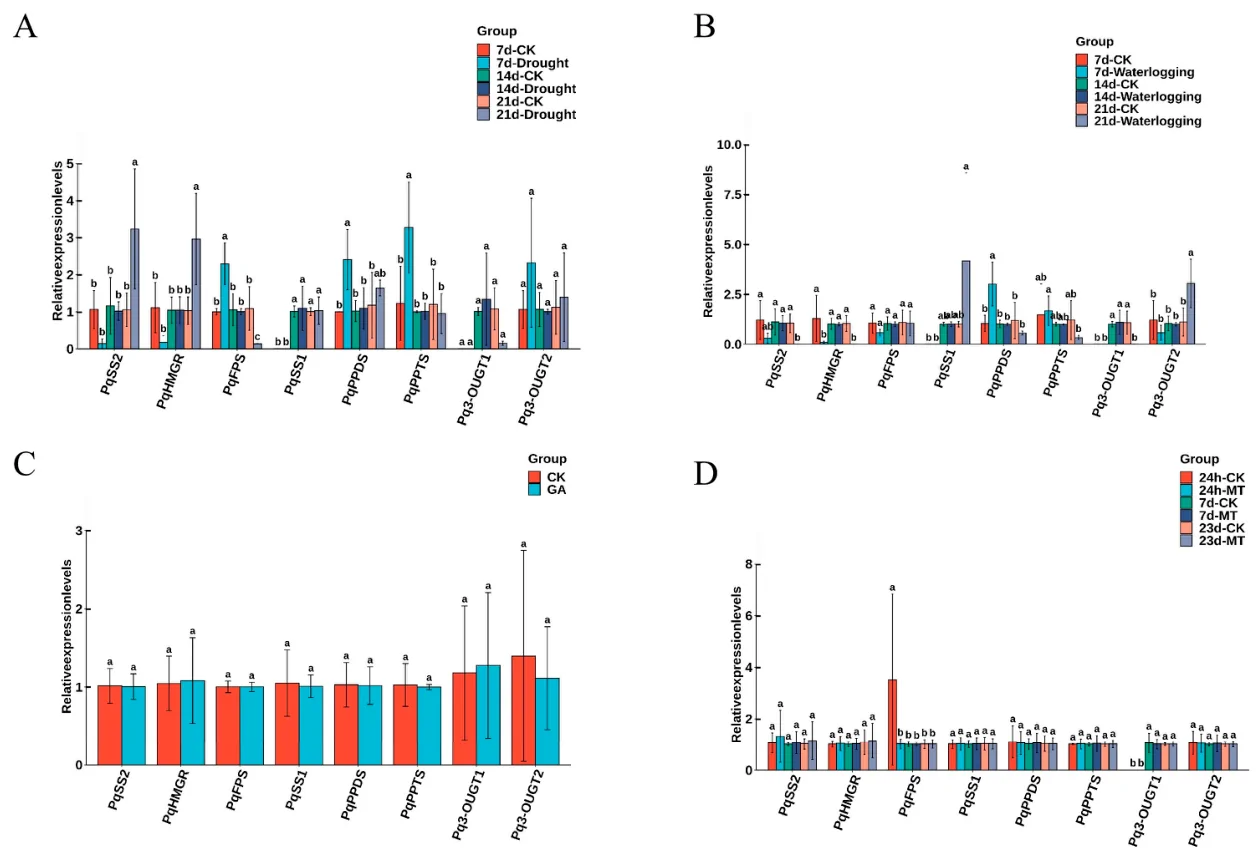
Relative expression analysis of ginsenoside biosynthesis-related genes in *Panax quinquefolius* under drought, waterlogging, gibberellin A_3_ (GA_3_), and melatonin (MT) treatments. (**A**) Drought stress (7d-CK, 7d-Drought, 14d-CK, 14d-Drought, 21d-CK, 21d-Drought). (**B**) Waterlogging stress (7d-CK, 7d-Waterlogging, 14d-CK, 14d-Waterlogging, 21d-CK, 21d-Waterlogging). (**C**) GA_3_ treatment (CK vs. GA). (**D**) MT treatment (24h-CK, 24h-MT, 7d-CK, 7d-MT, 23d-CK, 23d-MT). Each bar represents the mean ± standard error (SE). Different lowercase letters indicate significant differences (*p* < 0.05) between groups, as assessed by one-way ANOVA and Tukey’s multiple comparison test.

To validate the practical accuracy of the selected RGs, the expression patterns of eight key genes involved in the MVA-mediated ginsenoside biosynthetic pathway were quantified using the *UBC28* and *EF1-α* as reference genes (Tables S6 and S7, Figures S3 and S4). Under drought stress, the transcript levels of *PqSS2*, *PqHMGR*, *PqSS1*, *PqPPDS*, and *PqPPTS* were significantly up-regulated, as seen in Figure 5A. In contrast, waterlogging stress significantly induced the expression of *PqSS1*, *PqPPDS*, and *Pq3-OUGT2*, while significantly suppressing the expression of *PqSS2*, *PqHMGR*, and *Pq3-OUGT1*, as seen in Figure 5B. Neither exogenous GA_3_ nor melatonin treatments significantly affected the transcriptional changes in any of the eight ginsenoside biosynthesis genes under the tested concentration and treatment durations. Notably, extremely large differences in transcript abundance were detected under certain treatments. For example, the expression level of *PqSS1* was markedly elevated following 21-day waterlogging (Figure 5B), while an unusually high transcript level of *PqFPS* was observed in the 24 h-CK group (Figure 5D). These stress-responsive transcriptional changes are consistent with previously reported metabolic adaptations to water stress in plants, supporting the accuracy and reliability of the selected reference genes for qPCR normalization in *P. quinquefolius* [3,39].

Notably, neither exogenous GA_3_ nor melatonin treatments significantly affected the transcriptional changes in any of the eight ginsenoside biosynthesis genes under the tested concentration and treatment durations. These results suggest that short-term exogenous GA_3_ or melatonin is insufficient to substantially modulate the basal transcriptional activity of ginsenoside biosynthesis genes in *P. quinquefolius*.

## 3. Discussion

qRT-PCR is the most widely used technique for plant gene expression analysis, but its accuracy largely depends on the use of reliable reference genes for data normalization [40]. In non-model plant species, candidate reference genes are usually identified from orthologous sequences of well-characterized housekeeping genes in model plants owing to insufficient genetic and genomic sequence resources. However, numerous studies have demonstrated that genes proven to be stable reference genes in other plant species or experimental systems cannot be arbitrarily adopted for qRT-PCR normalization before their stability is validated under the specific experimental setup [24,25,26,27,28,29]. Relying exclusively on these classic housekeeping genes for qRT-PCR normalization therefore involves potential risks. Prior to this study, no systematically validated RGs were available for *P. quinquefolius*, limiting reliable gene expression studies related to ginsenoside biosynthesis and stress responses. Here, we systematically identified and validated both universal stable and condition-specific RGs for future functional genomic studies in American ginseng.

### 3.1. Advantages of the Candidate RG Screening Strategy

This study adopted a multi-dimensional rigorous screening strategy that integrated transcriptome-wide expression profiling with functional annotation. FPKM-based screening criteria enabled the selection of candidate genes with relatively high expression levels and stable basal transcription levels, thereby reducing the likelihood of including low- abundance transcripts with unstable quantification. Combined with the updated ICG 2.0 database annotation, we effectively identified the genes with conserved housekeeping functions across species, providing a rational basis for candidate gene selection [23,41]. Compared with the traditional random selection of classic RGs, this data-driven strategy reduces selection bias and improves the reliability of the candidate reference-gene set.

In addition, the SNP-based primer design strategy helped address non-specific amplification caused by highly homologous paralogous genes in the ginseng genome. Following the MIQE guidelines [22], we systematically optimized annealing temperature, primer concentration, and template input. This optimization improved amplification specificity and reproducibility. It also minimized technical variation in qRT-PCR and laid a solid foundation for subsequent reference-gene stability assessment and target-gene expression analysis.

### 3.2. Mechanistic Analysis of Differential RG Stability

Significant differences in expression stability were observed among candidate RGs under different experimental conditions, consistent with previous reports in *P. ginseng* and other medicinal plants. *UBC28* and *EF1-α* are involved in ubiquitin-mediated protein modification and protein translation, respectively, whereas ribosomal protein-coding genes such as *RPS5* participate in plant stress adaptation and translational regulation. We speculate that these functional differences may contribute to the observed variation in gene expression stability; however, further investigation is required to validate the underlying regulatory mechanisms.

We detected higher Ct values under long-term waterlogging. This pattern may indicate decreased transcript abundance in hypoxic root tissues. Although large-scale RNA degradation can be excluded for waterlogging samples from Figure S5, minor RNA fragmentation triggered by prolonged abiotic stress cannot be completely ruled out. Further transcriptome-wide data are needed to verify global transcriptional shifts. These findings further explain why a single constitutive reference gene cannot reliably normalize gene expression across diverse stress conditions, highlighting the necessity of identifying stress-specific optimal RGs. Although slight differences in gene stability rankings were observed among the various algorithms, these discrepancies mainly reflect their distinct statistical principles. By integrating the outputs of multiple algorithms, RefFinder minimizes the bias associated with individual methods and provides a more robust and comprehensive assessment of reference-gene stability [38].

Within the *Panax* genus, optimal reference genes exhibit both shared features and considerable inter-species divergence. In *P. ginseng*, translation-associated genes *EF1-γ*, *IF3G1*, and *EF1-β* ranked as the most stable across diverse tissues, while *IF3G1*, *ACT11*, and *GAPDH* performed best under heat-stress conditions [33]. In the present study on *P. quinquefolius*, *UBC28* and *EF1-α* were validated as the optimal reference genes for multiple tissues as well as drought, waterlogging, gibberellin A_3_ (GA_3_), and melatonin treatments. These findings highlight that even closely related Panax species lack universal reference genes. Accordingly, systematic experimental validation is mandatory for each species under its specific experimental conditions [33].

### 3.3. Biological Implication of Ginsenoside Biosynthesis Gene Responses

Ginsenoside biosynthesis is the major secondary metabolic pathway responsible for the pharmacological properties of American ginseng. In this study, drought and waterlogging strongly modified expression profiles of key genes in the mevalonate (MVA) pathway. This result indicates that soil water availability regulates ginsenoside biosynthesis at the transcriptional level. Previous studies have shown that water stress markedly affects ginsenoside accumulation in *P. quinquefolius*, although the underlying molecular mechanisms remain incompletely understood [42]. Our results provide molecular evidence linking transcriptional changes in biosynthetic genes to these physiological responses. These data advance our understanding of how soil moisture modulates ginsenoside metabolism under field conditions.

In contrast, exogenous GA_3_ and melatonin treatments showed no significant regulatory effects on the transcription of the tested ginsenoside biosynthesis genes under the tested conditions. It should be emphasized that our evaluation of reference-gene stability is entirely based on the expression consistency of reference genes themselves and does not rely on transcriptional changes in target genes. Although statistical analyses confirmed that the selected reference genes were stably expressed under hormone treatments, we lack scenarios with obvious target-gene transcriptional perturbation to further test the performance of reference genes in correcting large-scale biological expression variation. Multiple non-mutually exclusive factors may account for this observation. First, GA_3_ and melatonin may shape secondary metabolism via indirect signaling, post-transcriptional, or post-translational mechanisms, instead of directly modulating transcription of biosynthetic genes. Second, hormone responses frequently depend on tissue type and developmental stage, and such factors can also modify final regulatory outputs. Third, we cannot completely rule out the possibility that the concentrations and treatment durations applied in this study were inadequate to trigger detectable transcriptional responses. Therefore, further studies incorporating gradient hormone treatments, multiple developmental stages, and integrated transcriptomic, proteomic and metabolomic analyses will be necessary to elucidate the regulatory mechanisms by which these hormones influence ginsenoside biosynthesis.

### 3.4. Study Limitations and Future Prospects

This study established a standardized RG system for *P. quinquefolius* via qRT-PCR analysis for the first time, providing reliable internal control resources for subsequent functional gene research. Nevertheless, this study has several limitations. We evaluated reference-gene stability only under representative water stress and hormone treatments. Their performance under other conditions, including heat stress, salinity, and biotic stress, requires further validation. Moreover, enlarging the candidate gene set may help identify reference genes with higher stability and wider applicability.

Future work should test these optimal reference-genes across more biological contexts. It should also deploy them to dissect molecular mechanisms of ginsenoside biosynthesis under environmental and hormonal cues. Refining reference gene screening and validation pipelines will further improve the accuracy and reproducibility of qPCR assays for *P. quinquefolius* and other medicinal plants.

## 4. Materials and Methods

### 4.1. Plant Materials and Stress Treatments

*Panax quinquefolius* L. cv. LYS1 were grown at the Institute of Industrial Crops, Shandong Academy of Agricultural Sciences. For tissue-specific sampling, healthy 3-year-old plants with uniform growth were selected. Roots, stems, leaves, flowers, and fruits were collected separately with three biological replicates (three independent plants per replicate). All samples were gently cleaned to remove surface debris, immediately frozen in liquid nitrogen, and stored at −80 °C for subsequent RNA extraction. Four independent treatments were conducted in this study, including two abiotic stress treatments (drought, waterlogging) and two hormone treatments (GA_3_, melatonin). For the water stress treatments, three soil relative water content (RWC) levels were established: 30% RWC (severe drought stress), 70% RWC (untreated control), and 100% RWC (waterlogging stress). For the waterlogging group, a water layer (1–2 cm) was maintained on the soil surface. Each treatment contained three biological replicates. Soil moisture was gradually adjusted to the target level within 24 h to minimize environmental fluctuations. Root tissues were collected at 7, 14, and 21 days after treatment initiation. Soil RWC was monitored daily using a soil moisture sensor (Delta-T, Cambridge, UK) to ensure consistent treatment conditions throughout the experiment.

For melatonin (MT) treatment, uniform 3-year-old *P. quinquefolius* plants were foliar-sprayed with either deionized water (control) or 300 µmol/L MT solution. Spraying was performed three times at 5-day intervals to ensure sufficient MT uptake, with leaves sprayed uniformly until the surface was lightly moistened. Root samples were collected at 1, 7, and 23 days after the final treatment. For GA_3_ treatment, dormant buds collected from 2-year-old *P quinquefolius* plants were rinsed with sterile distilled water. Buds were inverted and fully immersed in 200 mg/L GA_3_ aqueous solution for 2 h. For the parallel control group, dormant buds were subjected to identical manipulation but soaked in sterile distilled water instead of GA_3_ solution. After soaking, aliquots of buds from both GA_3_ treated and control groups were immediately sampled to serve as the 0 h time point. The remaining buds were wrapped with sterile moist sphagnum moss and incubated under continuous darkness at 24 °C for 3 days prior to tissue collection.

All control and treatment samples were collected with three independent biological replicates, immediately snap-frozen in liquid nitrogen, and stored at −80 °C to prevent RNA degradation before total RNA extraction.

### 4.2. Total RNA Extraction and cDNA Synthesis

Total RNA was extracted from 100 mg frozen tissue powder using TRIzol reagent (Invitrogen, Carlsbad, CA, USA). Residual genomic DNA was removed via RNase-free DNase I (Takara, Dalian, China) treatment. RNA purity and concentration were measured using a NANO-200 ultramicro nucleic acid analyzer (AoSheng, Hangzhou, China). RNA integrity was assessed by 1% agarose gel electrophoresis (Figure S5). High-quality RNA samples with A260/280 ratios of 1.8–2.2 and A260/230 ratios ≥ 2.0 were used for first-strand cDNA synthesis. First-strand cDNA was synthesized using 1 µg qualified total RNA per sample with the First Strand cDNA Synthesis Kit (Thermo Scientific, Waltham, MA, USA) following the manufacturer’s standard protocol. The synthesized cDNA was stored at −20 °C for subsequent qRT-PCR assays.

### 4.3. Selection of Candidate Reference Genes

Candidate RGs were screened from multi-tissue RNA-Seq datasets of *P. quinquefolius*. Raw reads were trimmed using Trimmomatic and aligned to the American ginseng reference genome (PRJCA006678) from the National Genomics Data Center (NGDC, https://ngdc.cncb.ac.cn) via HISAT2 2.2.1. Gene expression levels were quantified as fragments per kilobase per million mapped reads (FPKM). Based on RNA-seq data from five tissues (root, stem, leaf, flower, fruit), each represented by three biological replicates, the mean FPKM value (MV), standard deviation (SD), maximum FPKM fold change (MFC), and coefficient of variation (CV = SD/MV) were calculated for each gene. Genes were preliminarily filtered using the following strict criteria: MV > 50, MFC < 1.5, CV < 0.2, and ranked by low SD values. Candidate RGs were further identified through homology searches against the ICG 2.0 database and alignment comparison with well-characterized RGs in *Arabidopsis thaliana*.

### 4.4. Primer Design for qRT-PCR

The cDNA and protein sequences of candidate genes were retrieved from the NGDC Genome Warehouse. To minimize non-specific amplification caused by highly homologous gene copies, single-nucleotide polymorphism (SNP) loci between each target gene and their homologs were identified by multiple sequence alignment. Multiple sequence alignment was performed using ClustalX 2.0, and cDNA alignments were manually refined according to protein sequence conservation to ensure accuracy.

Gene-specific primers were preferentially designed using terminal SNP sites to guarantee amplification specificity. For each candidate RG, four pairs of specific primers (20–23 bp, GC content 40–60%) were designed, with target amplicons ranging from 85 to 125 bp. For genes exhibiting extremely high sequence similarity to paralogs, slightly longer amplicons were allowed to facilitate the design of highly primer specificity. All primer pairs were evaluated for no continuous base repeats and reasonable terminal base distribution to meet qRT-PCR amplification requirements.

### 4.5. Optimization of qRT-PCR Conditions

Optimization of qRT-PCR amplification conditions was performed following the protocol of Zhao et al. [20]. Gradient tests of annealing temperature (56.0–62.7 °C) and primer concentration (250–400 nM) were carried out individually for every primer set to identify the optimum reaction parameters. The optimal conditions were selected based on the lowest Ct value together with specific amplification, as confirmed by melting curve analysis. Serial two-fold cDNA dilutions (1:10 to 1:160) were used to generate standard curves. The amplification efficiency was calculated with the formula: E = (10^(−1/slope)^ − 1) × 100. Qualified primer pairs strictly met the criteria of coefficient of determination *R*^2^ ≥ 0.99 and amplification efficiency of 100 ± 5%, which is the mandatory prerequisite for the reliable quantitative method. Unqualified primer pairs with inconsistent linearity or abnormal amplification efficiency were eliminated to ensure experimental accuracy.

### 4.6. qRT-PCR Assay

Validated optimal primer pairs were used for qRT-PCR analysis of candidate RG expression profiles across all experimental groups. Each treatment included three biological replicates, with three technical replicates per biological replicate. The 10 µL qRT-PCR reaction system consisted of 5 µL SYBR Select Master Mix (Takara Bio, Dalian, China), 0.3 µL each of the forward primer and reverse primer, 1 µL diluted cDNA template, and 3.4 µL ddH_2_O. Reaction components except for the master mix were pre-mixed uniformly, centrifuged briefly, and dispensed into 96-well plates before adding the fluorescent master mix to ensure reaction consistency.

The qRT-PCR amplification program was set as follows: initial denaturation at 95 °C for 2 min; 39 cycles of 95 °C for 5 s and optimal annealing temperature for 30 s; and final extension at 65 °C for 5 s. Melting curve analysis (65–95 °C, 0.5 °C incremental increase) was performed after amplification to confirm single specific amplification peaks and exclude non-specific products and primer dimers.

### 4.7. Stability Analysis of Candidate Reference Genes

The expression stability of candidate reference genes was comprehensively evaluated using five widely adopted algorithms: ΔCt, geNorm, NormFinder, BestKeeper, and RefFinder. Briefly, lower SD values in ΔCt analysis, lower stability values in NormFinder, lower M values in geNorm, and lower CV/SD values in BestKeeper indicate higher expression stability. RefFinder integrates the rankings generated by these four algorithms and calculates a comprehensive geometric mean to provide an overall stability ranking, thereby minimizing the bias associated with individual evaluation methods and obtaining the most credible stability order.

### 4.8. Data Statistical Analysis

The relative expression levels of eight key ginsenoside biosynthesis genes in the MVA pathway were quantified using the standardized dual-reference-gene normalization algorithm [35,43]. Statistical significance was determined via one-way ANOVA followed by Duncan’s multiple range test, with *p* < 0.05 defined as statistically significant. All quantitative data were presented as mean ± standard error (SE).

## 5. Conclusions

In this study, 15 candidate RGs were systematically identified from *P. quinquefolius* transcriptome data and validated via qRT-PCR under five experimental conditions. *UBC28* and *EF1-α* were identified as the optimal reference-genes suitable for multi-tissue and multi-treatment qRT-PCR normalization, while condition-specific stable RGs were also determined for drought, waterlogging, GA_3_, and melatonin treatments. Functional validation using ginsenoside biosynthesis-related genes demonstrated that the selected RGs provide accurate reliable normalization for qRT-PCR analyses. Collectively, this study establishes a standardized reference gene framework for *P. quinquefolius*, providing an important methodological resource for future investigations of ginsenoside biosynthesis, stress response mechanisms, and other functional genomic studies in American ginseng.

## Figures and Tables

**Table 1:** The optimized qPCR conditions for the 15 candidate reference genes in American ginseng.

Primer Pair	Optimal Tm (°C)	Optimal Primer Concentration (mM)	cDNA Dilution	*R* ^2^	E (%)
**ALDH6B2-qPCR-F1/ALDH6B2-qPCR-R1**	**62.8**	**400**	**1/20–1/160**	**0.9932**	**100.5**
**Carmnt1-qPCR-F1/Carmnt1-qPCR-R1**	**62.8**	**400**	**1/20–1/80**	**1**	**92.3**
**EF1-α-qPCR-F1/EF1-α-qPCR-R1**	**62.8**	**400**	**1/10–1/40**	**0.9979**	**99.9**
EF1-α-qPCR-F1/EF1-α-qPCR-R2	62.8	400	1/10–1/40	0.9923	96.7
EIF4A3A-qPCR-F1/EIF4A3A-qPCR-R1	62.8	400	1/10–1/80	0.9995	95.5
**EIF4A3A-qPCR-F1/EIF4A3A-qPCR-R2**	**62.8**	**400**	**1/20–1/80**	**0.9556**	**99.7**
EIF4A3A-qPCR-F2/EIF4A3A-qPCR-R1	62.8	400	1/10–1/80	0.985	96.9
EIF4A3A-qPCR-F2/EIF4A3A-qPCR-R2	62.8	400	1/20–1/160	0.9673	90.8
RPL10-qPCR-F1/RPL10-qPCR-R1	56.2	400	1/10–1/80	0.9797	95.2
**RPL10-qPCR-F1/RPL10-qPCR-R2**	**56.2**	**400**	**1/10–1/40**	**0.9991**	**102.3**
RPL10-qPCR-F2/RPL10-qPCR-R1	56.2	400	1/10–1/80	0.9801	100.2
RPL10-qPCR-F2/RPL10-qPCR-R2	56.2	400	1/40–1/160	0.9998	89.3
**RPS5-1-qPCR-F1/RPS5-1-qPCR-R1**	**62.8**	**400**	**1/40–1/160**	**0.9932**	**100.1**
RPS5-1-qPCR-F2/RPS5-1-qPCR-R1	62.8	400	1/20–1/160	0.993	102.5
RPS5-2-qPCR-F1/RPS5-2-qPCR-R1	62.8	400	1/40–1/160	0.9842	101.1
**RPS5-2-qPCR-F1/RPS5-2-qPCR-R2**	**62.8**	**300**	**1/20–1/160**	**0.9993**	**100.3**
**SKP1A-2-qPCR-F1/SKP1A-2-qPCR-R1**	**62.8**	**400**	**1/40–1/160**	**0.9972**	**97.6**
SKP1A-2-qPCR-F1/SKP1A-2-qPCR-R2	62.8	300	1/20–1/160	0.9837	101.2
SKPIA-1-qPCR-F1/SKPIA-1-qPCR-R1	62.8	350	1/40–1/160	0.9371	91.6
SKPIA-1-qPCR-F1/SKPIA-1-qPCR-R2	62.8	350	1/20–1/160	0.9835	92.9
SKPIA-1-qPCR-F2/SKPIA-1-qPCR-R1	62.8	350	1/20–1/160	0.9883	93.7
**SKPIA-1-qPCR-F2/SKPIA-1-qPCR-R2**	**62.8**	**350**	**1/20–1/160**	**0.9986**	**100.3**
**EIF1AX-2-qPCR-F1/EIF1AX-2-qPCR-R1**	**62.8**	**350**	**1/40–1/160**	**0.9904**	**105.1**
**TCTP-qPCR-F1/TCTP-qPCR-R1**	**53.9**	**300**	**1/10–1/40**	**0.9922**	**100.1**
UBC2-1-qPCR-F1/UBC2-1-qPCR-R1	62.8	400	1/20–1/80	1	91.1
UBC2-1-qPCR-F1/UBC2-1-qPCR-R2	62.8	400	1/40–1/160	0.997	92
UBC2-1-qPCR-F2/UBC2-1-qPCR-R1	62.8	400	1/40–1/160	0.9582	105.9
**UBC2-1-qPCR-F2/UBC2-1-qPCR-R2**	**62.8**	**350**	**1/20–1/80**	**0.9313**	**101.1**
**UBC2-2-qPCR-F1/UBC2-2-qPCR-R1**	**62.8**	**350**	**1/20**–**1/80**	**0.9983**	**102.4**
UBC2-2-qPCR-F1/UBC2-2-qPCR-R2	62.8	350	1/20–1/160	0.9713	85.4
UBC2-2-qPCR-F2/UBC2-2-qPCR-R1	62.8	350	1/20–1/160	0.9961	83.5
UBC2-2-qPCR-F2/UBC2-2-qPCR-R2	62.8	400	1/20–1/160	0.9923	92
UBC28-qPCR-F1/UBC28-qPCR-R1	62.8	300	1/10–/40	0.9958	102.8
UBC28-qPCR-F1/UBC28-qPCR-R2	62.8	300	1/10–1/40	0.9974	107.4
UBC28-qPCR-F2/UBC28-qPCR-R1	62.8	300	1/10–1/160	0.9832	101
**UBC28-qPCR-F2/UBC28-qPCR-R2**	**62.8**	**300**	**1/20**–**1/160**	**0.9981**	**100.9**
RPL40e-qPCR-F1/RPL40e-qPCR-R1	62.8	400	1/10–1/40	0.9918	101
**RPL40e-qPCR-F1/RPL40e-qPCR-R2**	**62.8**	**400**	**1/10**–**1/40**	**0.9989**	**97.2**
RPL40e-qPCR-F2/RPL40e-qPCR-R1	62.8	400	1/10–1/40	0.9944	86.3
RPL40e-qPCR-F2/RPL40e-qPCR-R2	62.8	400	1/20–1/80	0.9974	84.4

The optimal primer pairs are shown in bold.

**Table 2:** Comprehensive stability ranking of candidate reference genes calculated by five different algorithms under various experimental-treatment conditions.

Gene	Ranking Order of Delta CT	Ranking Order of BestKeeper	Ranking Order of Normfinder	Ranking Order of geNorm	Ranking Order of RefFinder
*UBC28*	1	6	1	4	1
*EF1-α*	2	8	2	1	2
*RPL40e*	3	3	4	1	3
*Carnmt1*	4	10	3	3	4
*EIF1AX*	5	4	5	5	5
*UBC2-2*	8	2	8	6	6
*SKP1A-1*	6	5	6	7	7
*RPS5-2*	13	1	13	12	8
*RPL10*	7	11	7	8	9
*EIF4A3A*	10	7	10	10	10
*TCTP*	11	9	12	9	11
*SKP1A-2*	9	12	9	11	12
*ALDH6B2*	12	13	11	13	13
*RPS5-1*	14	15	14	14	14
*UBC2-1*	15	14	15	15	15

Green and red shading indicate the most stable and least stable candidate reference genes, respectively, as evaluated by each algorithm.

**Table 3:** Ranking of expression stability of the 15 candidate reference genes in American ginseng with Ct values being calculated using RefFinder.

Ranking Order	Tissues	Drought	Waterlogging	Water Stress	MT	GA	Hormones
1	*RPS51*	*UBC28*	*UBC28*	*RPL40e*	*EF1-α*	*UBC2-1*	*EF1-α*
2	*TCTP*	*RPS52*	*ALDH6B2*	*UBC28*	*RPS52*	*ALDH6B2*	*RPS51*
3	*RPS52*	*EIF1AX*	*RPL40e*	*RPS5-2*	*RPL40e*	*SKP1A-1*	*SKP1A-1*
4	*ALDH6B2*	*RPL40e*	*RPS52*	*ALDH6B2*	*EIF4A3A*	*EF1-α*	*RPL40e*
5	*RPL10*	*ALDH6B2*	*EIF1AX*	*EIF1AX*	*RPS51*	*RPL10*	*UBC28*
6	*Carnmt1*	*RPL10*	*EF1-α*	*EF1-α*	*ALDH6B2*	*Carnmt1*	*EIF4A3A*
7	*RPL40e*	*TCTP*	*RPL10*	*Carnmt1*	*UBC28*	*RPL40e*	*SKP1A-2*
8	*UBC2-2*	*UBC2-1*	*Carnmt1*	*UBC2-1*	*SKP1A-1*	*RPS52*	*Carnmt1*
9	*EF1-α*	*SKP1A-2*	*SKP1A-1*	*RPL10*	*UBC2-2*	*SKP1A-2*	*UBC2-2*
10	*UBC28*	*EF1-α*	*UBC2-1*	*TCTP*	*SKP1A-2*	*EIF1AX*	*UBC2-1*
11	*EIF1AX*	*Carnmt1*	*TCTP*	*SKP1A-1*	*Carnmt1*	*RPS51*	*EIF1AX*
12	*EIF4A3A*	*SKP1A-1*	*UBC2-2*	*UBC2-2*	*EIF1AX*	*UBC28*	*RPL10*
13	*SKP1A-1*	*EIF4A3A*	*EIF4A3A*	*SKP1A-2*	*UBC2-1*	*UBC2-2*	*ALDH6B2*
14	*SKP1A-2*	*UBC2-2*	*SKP1A-2*	*EIF4A3A*	*RPL10*	*EIF4A3A*	*RPS52*
15	*UBC2-1*	*RPS51*	*RPS51*	*RPS5-1*	*TCTP*	*TCTP*	*TCTP*

Green and red backgrounds indicate the most stable and least stable genes according to the comprehensive ranking of RefFinder, respectively.

## Data Availability

The data presented in this study are available upon request from the corresponding authors.

## Supplementary Materials

The following supporting information can be downloaded at: https://www.mdpi.com/article/10.3390/plants15182778/s1, Figure S1: Ranking of expression stability for 15 candidate reference genes using four algorithms (NormFinder, delta-CT method, geNorm, and BestKeeper) and the comprehensive ranking generated by RefFinder. Figure S2: Comprehensive stability ranking of candidate reference genes under different experimental conditions (drought, GA, tissues, waterlogging, MT, water stress, and hormones) based on RefFinder. Figure S3: Melting curves of eight key genes involved in the MVA-mediated ginsenoside biosynthetic pathway in qPCR assays for primer specificity verification. Figure S4: Standard curves of eight key genes involved in the MVA-mediated ginsenoside biosynthetic pathway showing the linear relationship between Ct values and log_10_-transformed cDNA template concentration. Figure S5: Agarose gel electrophoresis of total RNA from American ginseng under drought, waterlogging, and hormone treatments. Table S1: Expression levels of 15 candidate reference genes in five different tissues of American ginseng. Table S2: Primer pairs of the 15 candidate reference genes. Table S3: Transcript abundance of 15 selected candidate genes. Table S4: The expression stability values of 15 selected reference genes across samples determined via different evaluation algorithms: RefFinder, delta-CT, BestKeeper, NormFinder, and geNorm. Table S5: The expression stability values of 15 candidate reference genes in American ginseng being calculated using RefFinder. Table S6: Primer pairs of the eight ginsenoside biosynthesis-related genes in American ginseng. Table S7: The optimized qPCR conditions for the eight ginsenoside pathway genes in American ginseng.
